# CuO hollow nanosphere-catalyzed cross-coupling of aryl iodides with thiols

**DOI:** 10.1186/1556-276X-8-390

**Published:** 2013-09-17

**Authors:** Hyunje Woo, Balaji Mohan, Eunjung Heo, Ji Chan Park, Hyunjoon Song, Kang Hyun Park

**Affiliations:** 1 Department of Chemistry and Chemistry Institute for Functional Materials, Pusan National University, Busan 609-735, Korea; 2 Clean Fuel Department, Korea Institute of Energy Research, Daejeon 305-343, Korea; 3 Department of Chemistry, Korea Advanced Institute of Science and Technology, Daejeon 305-701, Korea

**Keywords:** Microwave, Copper oxide, Acetylene black, Heterogeneous, Ullmann

## Abstract

New functionalized CuO hollow nanospheres on acetylene black (CuO/AB) and on charcoal (CuO/C) have been found to be effective catalysts for C-S bond formation under microwave irradiation. CuO catalysts showed high catalytic activity with a wide variety of substituents which include electron-rich and electron-poor aryl iodides with thiophenols by the addition of two equivalents of K_2_CO_3_ as base in the absence of ligands.

## Background

Sulfur-containing aromatic compounds, notably aryl sulfides and their derivatives, are prominent in fields such as biological, pharmaceutical, and materials fields. In particular, their use in synthesizing biologically and pharmaceutically important organosulfur compounds such as HIV protease inhibitors [1] (Viracept, Nelfinavir Mesylate, AG 1343), LFA-1/ICAM-1 antagonists [2], and arylthioindoles [3] (potent inhibitors of tubulin assembly) is still not fully understood by synthetic chemists. In general, molecules containing one or more carbon-sulfur bonds can be used as molecular precursors for the synthesis of new materials [4]. However, compared to C-N and C-O bonds, the transition metal-catalyzed C(aryl)-S bond formation has not been well studied. This bond formation is thought to be partial because of the formation of an S-S coupled product and a concurrent deactivation of the metal catalyst due to the strong coordinative and adsorptive properties of sulfur, which can decrease catalytic activity [5]. General methods for C-S cross-coupling involve the condensation of aryl halides with thiols and, usually, require temperatures greater than 200°C. These methods also require strongly basic, toxic, high-boiling, polar solvents, namely HMPA, quinolone, or *N*,*N*-dimethylacetamide. In order to circumvent these complications, a meticulous effort has been focused on the development of transition metal-catalyzed coupling of thiophenols with aryl halides. Previously, iron [6], nickel [7,8], palladium [9,10], cobalt [11], and copper-based [12-16] catalytic systems have been reported for this purpose. Even though significant improvements have been made, appropriate techniques are still needed for the synthesis of diaryl thioethers. To date, metal and metal oxide nanoparticles have often been used as metal catalysts because of their physical and chemical stability. In addition, the advantage of nanoparticles including large surface area and heterogeneous nature make them applicable to a broad range of scientific fields and functions such as the immobilization of biomolecules [17], catalysis of organic [18-23] and electrochemical reactions [17], use in electrochemical sensors and biosensors [17], enhancement of electron transfer [17], labeling of biomolecules [17], and synthesis of nanofluids [24], antibacterial materials [25], photocatalysts [25,26], solar cells [27], and so on. Among the various available metal oxide nanoparticles, two copper oxides (Cu_2_O, CuO) have been studied for use in p-type semiconductor materials with narrow band gaps. This is because copper oxides are less expensive, recyclable, and non-toxic and have suitable optical and electronic properties [28-32]. Thus, as part of the effort to find new catalytic systems and better understand the role of transition metal nanoparticles in organic transformations, we report herein the use of CuO hollow nanoparticles as catalysts for efficient syntheses of diaryl thioethers. These CuO hollow nanoparticles have advantages in terms of large-scale synthesis and uniform shape compared to previous reported CuO nanoparticles [33,34]. In recent times, microwave-irradiated organic reactions have become increasingly popular as valuable alternatives to the use of conductive heating for promoting chemical reactions. Besides, improved yields within short reaction time were observed. Microwave activation, as a non-conventional energy source, is becoming a very popular and valuable technique in organic synthesis, as evidenced by the increasing number of annual publications on this topic. In continuation of our previous reports [35], we discovered that microwave irradiation can even accelerate the Ullmann coupling of activated aryl iodides and thiophenols.

## Methods

### General

Reagents were purchased from Aldrich Chemical Co. (St. Louis, MO, USA) and Strem Chemical Co. (Bischheim, France) and used as received. Reaction products were analyzed by the literature values of known compounds. CuO, CuO/AB, and CuO/C were characterized by transmission electron microscopy (TEM) (Philips F20 Tecnai operated at 200 kV, KAIST, Amsterdam, the Netherlands). Samples were prepared by placing a few drops of the corresponding colloidal solution on carbon-coated copper grids (Ted Pellar, Inc., Redding, CA, USA). The X-ray diffractometer (XRD) patterns were recorded on a Rigaku D/MAX-RB (12 kW; Shibuya-ku, Japan) diffractometer. The copper loading amounts were measured by inductively coupled plasma atomic emission spectroscopy (ICP-AES). Elemental compositions of CuO/AB were obtained using energy-dispersive X-ray spectroscopy (EDS) (550i, IXRF Systems, Inc., Austin, TX, USA).

### Preparation of Cu_2_O nanocubes

Poly(vinylpyrrolidone) (PVP, Aldrich, M_w_ 55,000; 5.3 g), dissolved in 45 mL of 1,5-pentanediol (PD, Aldrich, 96%), was heated to 240°C under inert conditions. Then, 4.0 mmol of Cu(acac)_2_ (Strem, 98%), dissolved in 15 mL of PD, was injected into the hot PVP solution at 240°C, and the mixture was stirred for 15 min at the same temperature. The resulting colloidal dispersion was cooled to room temperature, and the product was separated by adding 150 mL of acetone, with centrifugation at 8,000 rpm for 20 min. The precipitates were washed with ethanol several times and re-dispersed in 50 mL of ethanol.

### Synthesis of CuO hollow nanostructures

An appropriate concentration of aqueous ammonia solution was added to 25 mL of the Cu_2_O cube dispersion in ethanol (16 mM with respect to the precursor concentration). The mixture was subjected to stirring at room temperature for 2 h. The volume and concentration of the aqueous ammonia solution used for each structure were 1.0 mL and 14.7 M, respectively, for hollow cubes; 2.0 mL and 7.36 M, respectively, for hollow spheres; and 6.0 mL and 2.45 M for urchin-like particles, respectively. For shape optimization of the hollow spheres, a 3.68-M aqueous ammonia solution was used. After the reaction, the products were collected by centrifugation at 6,000 rpm for 20 min.

### Synthesis procedures of CuO/AB and CuO/C

The acetylene carbon black (STREM, 99.99%, 1.2 g) was mixed with 100 mL of the CuO hollow nanosphere dispersion in ethanol (17.0 mM), and the reaction mixture was sonicated for 1 h at room temperature. After 1 h, the product CuO/AB was washed with ethanol several times and vacuum dried at room temperature. For the synthesis of CuO/C, the mixture solution of charcoal (0.8 g) and 50.0 mL of CuO hollow nanosphere dispersion in ethanol (50.0 mM) was refluxed for 4 h. After 4 h, the black suspension was cooled to room temperature and precipitated by centrifugation. The product CuO/C was washed with ethanol thoroughly and dried in a vacuum oven at room temperature.

### General procedure for cross-coupling of aryl halides with thiophenol

Into a 10-mL glass vial, 4.0 mg of CuO/AB and CuO/C, iodobenzene (0.11 mL, 1.0 mmol), thiophenol (0.11 mL, 1.1 mmol), and solvent (5.0 ml) were placed. The reaction mixture was irradiated with a microwave stove (MAS II, Sineo Microwave Chemistry Technology Co., Ltd., Shanghai, China) for 10 to 30 min. After reaction, the vial was cooled to RT. The solution was then filtered, concentrated under reduced pressure, and characterized by Gas chromatography–mass spectrometry (GC-MS) spectra. Yields were based on the amount of iodobenzene used in each reaction.

## Results and discussion

### Catalyst characterization

**Figure 1:**
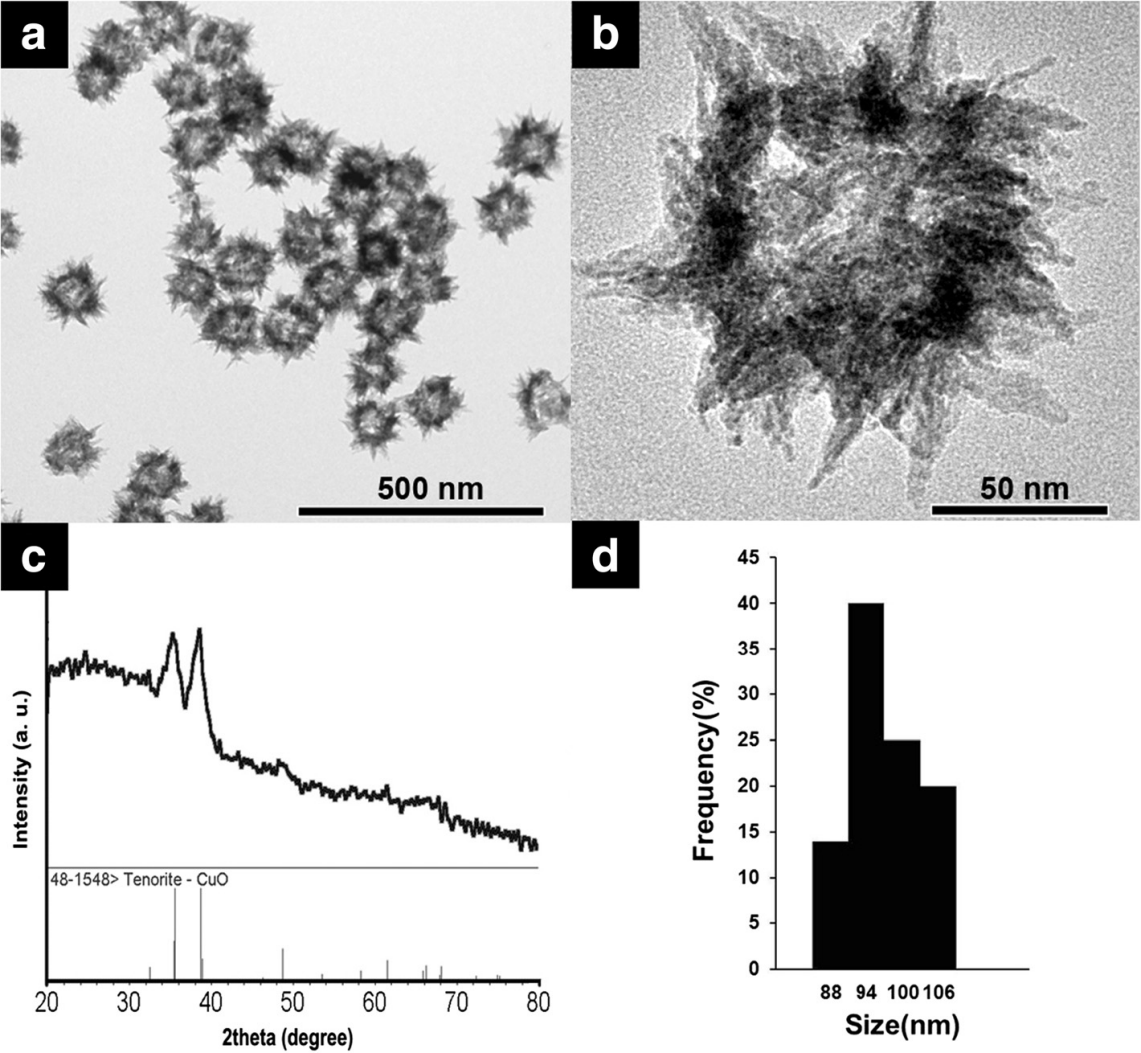
TEM images of (a, b) CuO hollow nanospheres; (c) XRD pattern; (d) size distribution diagram of CuO hollow nanospheres.

The CuO hollow nanostructures were prepared by a controlled oxidation of Cu_2_O nanocubes using an aqueous ammonia solution according to a method in the literature [36]. The Cu_2_O nanocubes (average edge size of 50 nm) were converted to CuO hollow nanospheres by addition of ammonia solution (2.0 mL, 3.7 M) into Cu_2_O colloidal solution by a dissolution-precipitation process. The TEM images in Figure 1a,b show monodisperse CuO hollow nanospheres that are composed of needle-like branches. The average size of these CuO hollow nanospheres was measured to be 103 ± 8 nm (Figure 1d). The CuO hollow nanospheres were analyzed using XRD analysis (Figure 1c). Two main peaks were present in the XRD patterns of the CuO hollow nanospheres that could be assigned to the reflections of the (002)/(11–1) and (111)/(200) planes in the CuO phase (JCPDS no. 48–1548).

**Figure 2:**
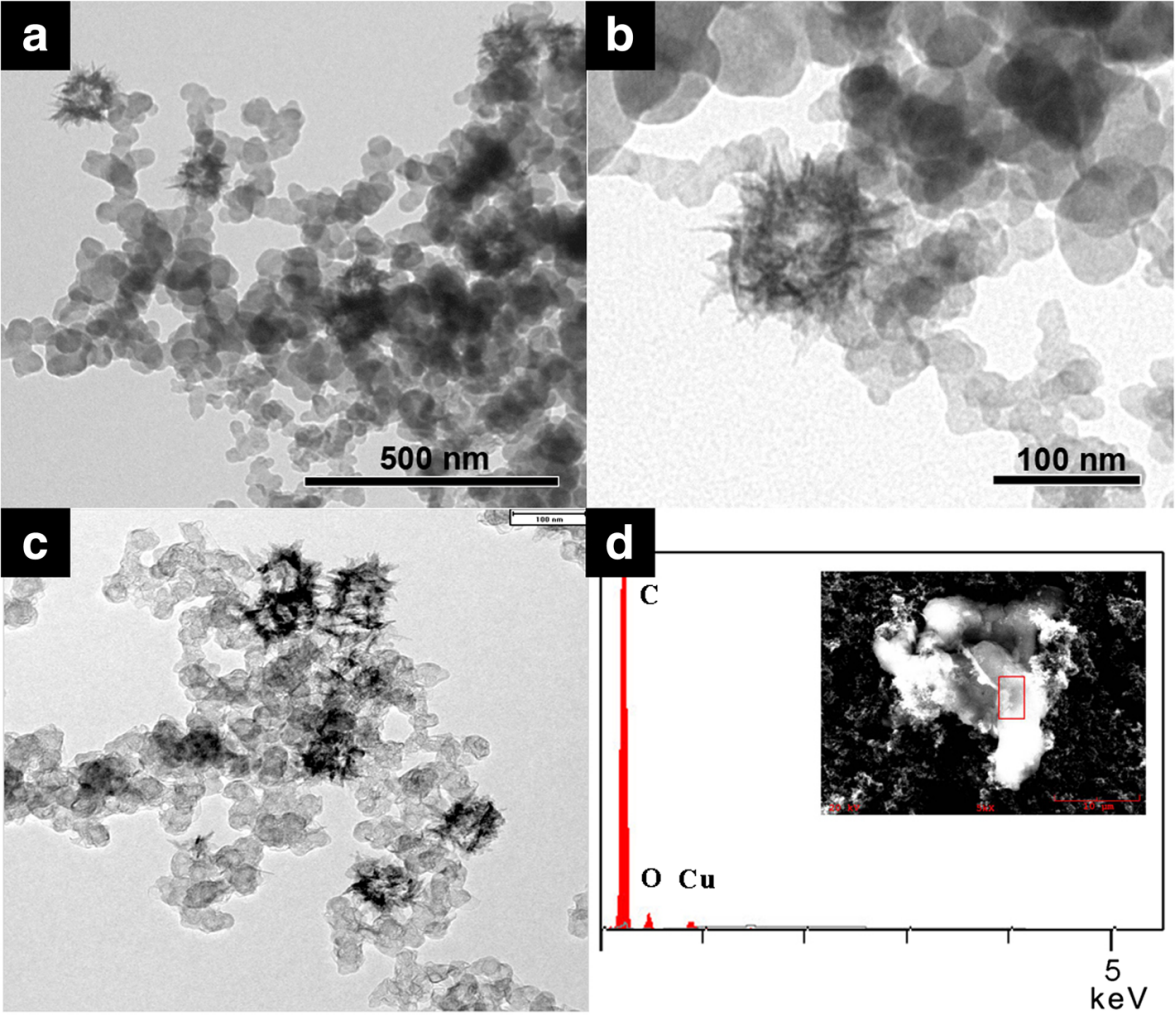
**TEM images and EDS spectrum.** TEM images of **(a**, **b)** CuO/AB. TEM image of **(c)** CuO/C, and the scale bar represents 200 nm. EDS spectrum of **(d)** CuO/AB.

Immobilization of CuO hollow nanospheres on acetylene black (CuO/AB) was performed by sonication for 1 h at room temperature. The TEM images in Figure 2a,c show well-dispersed CuO/AB and CuO/C, maintaining their original size and structure. ICP-AES confirmed the content of copper metal on the acetylene black. EDS spectrum in Figure 2d showed that hollow CuO nanoparticles were immobilized on acetylene black. The X-ray photoelectron spectroscopy data at the energy regions of the Cu bands confirm that the elements of the three different shapes are only Cu(II). The peaks at 933.8 and 953.7 eV correspond to Cu 2p_3/2_ and Cu 2p_1/2_ bands, and the other two signals, at 943.8 and 962.4 eV, are the shakeup satellites, which are characteristic of d^9^ Cu(II) compounds [37].

### Ullmann reaction of aryl halides with thiols catalyzed by CuO hollow nanoparticles

**Scheme 1:**
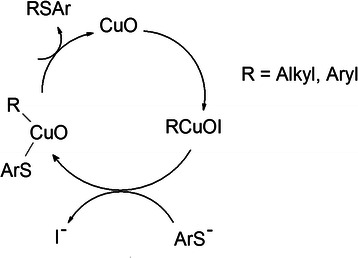
Proposed mechanism for synthesis of aryl thioethers.

**Figure 3:**
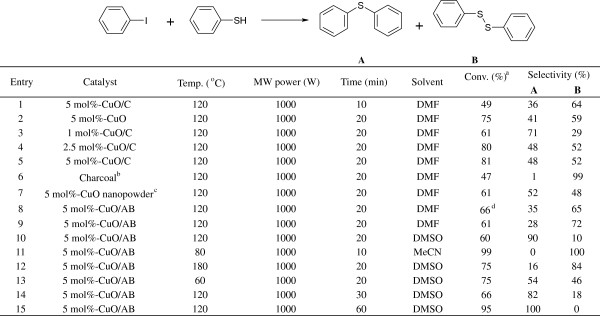
Ullmann coupling reaction of iodobenzene with thiophenol.

Initially, the reaction of iodobenzene with thiophenol was chosen as a model reaction. Reaction mechanism about Ullmann coupling is already reported [38]. Scheme 1 shows a proposed mechanism for synthesis of aryl thioethers. To optimize the reaction, several experiments were performed by varying solvent, reaction time, and reaction temperature and using either hollow nanospherical CuO, CuO/C, or CuO/AB as the catalyst. First, 5.0 mol% of hollow nanospherical CuO/C in DMF were used at a temperature of 120°C, and diphenyl thioether was obtained with 49% conversion (entry 1, Figure 3). CuO hollow nanoparticles were used as a catalyst to compare the catalytic activity with supported CuO catalysts and showed 75% conversion (entry 2, Figure 3). Quantity of catalyst was also checked to observe the catalytic activity of CuO/C catalyst. There was no difference in conversion between 2.5 and 5 mol% of the catalyst (entries 3 to 5, Figure 3). When the reaction time was increased to 20 min, 81% conversion was achieved under the same conditions but with slight deviation in selectivity (entry 5, Figure 3). Only charcoal catalyst showed less catalytic activity and selectivity (entry 6, Figure 3). We tried one reaction using commercially available CuO nanopowder as catalyst. CuO nanopowder exhibited less catalytic activity than CuO/C catalyst although there is no surfactant in CuO nanopowder (entries 5 and 7, Figure 3). Our CuO hollow nanostructure showed better catalytic activity because of a high surface area. Conversion of 66% was achieved with the use of two equivalent thiophenols (2.2 mmol), and the amount of diphenyl disulfide increased due to homocoupling reaction as expected (entry 8, Figure 3). Next, the catalytic activity of the hollow nanospherical CuO/AB was compared with that of the hollow nanospherical CuO/C catalyst at the same condition. The catalytic activities of both catalysts were almost equivalent, and 61% conversion was obtained (entry 9, Figure 3). Interestingly, when the solvent was changed to dimethyl sulfoxide (DMSO), diphenyl thioether was dominant under the same conditions (entry 10, Figure 3). At a temperature of 80°C and a reaction time of 10 min, >% conversion of diphenyl disulfide was achieved in the presence of MeCN (entry 11, Figure 3). There was no difference in the conversion between reaction temperatures of 180°C and 60°C (entries 12 and 13, Figure 3). When the reaction time was increased to 30 min, the conversion was slightly increased and the selectivity of diphenyl thioether was decreased (entry 14, Figure 3). We found that selectivity was dependent on several factors such as solvent used (entries 9 to 11, Figure 3), quantity of thiophenol (entries 8 and 9, Figure 3), reaction temperature (entries 12 to 14, Figure 3), and reaction time (entries 10 and 14, Figure 3).

**Figure 4:**
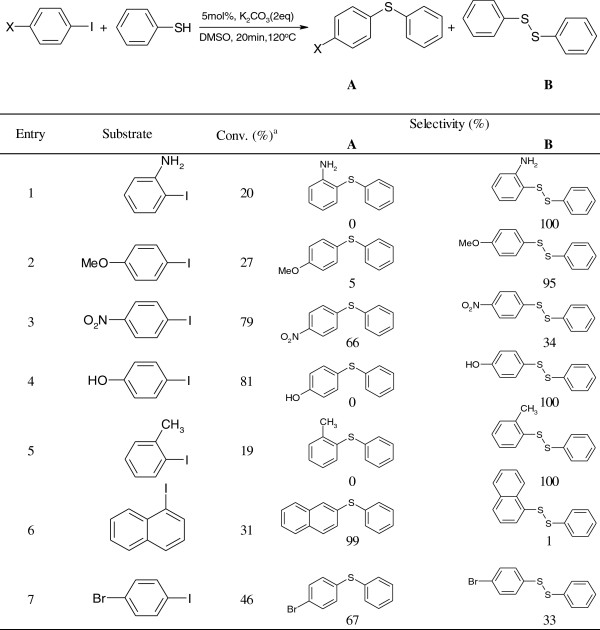
CuO/AB-catalyzed Ullmann coupling reaction with various substrates.

The versatilities of our nanocatalyst were investigated by performing Ullmann coupling reactions of various substrates under optimized reaction conditions. The reactions of substrates with electron-rich and electron-poor groups on the iodobenzene resulted in different yields and selectivities of the cross-coupling products (Figure 4). When the electron-rich substrates were used, more than 95% selectivity for diphenyl disulfide was obtained due to a homocoupling reaction of thiophenol although only a low yield of product was obtained in this case (entries 1, 2, 4, and 5, Figure 4). On the contrary, only 79% conversion was obtained in the case of electron-poor substituents such as 1-iodo-4-nitro-benzene, and the selectivity for product (A) was increased to 66% (entry 3, Figure 4). Interestingly, the reaction of substrates with -NO_2_ group was found to have high selectivity on product (A) although it had a low conversion (entry 6, Figure 4). A regioselectivity test was performed using thiophenol and 1-bromo-4-benzene. 4-Bromo diphenyl sulfide (selectivity of 100%) was formed with 46% conversion.

## Conclusions

In conclusion, CuO hollow nanospheres were synthesized by controlled oxidation of Cu_2_O nanocubes using aqueous ammonia solutions. Ullmann coupling reactions of aryl iodide with thiols were conducted to check the respective catalytic activities of CuO, CuO/AB, and CuO/C hollow nanosphere catalysts under microwave irradiation. Various diaryl thioethers were obtained from electron-deficient aryl iodides, while diaryl disulfide was produced from electron-rich aryl iodides. Transition metals loaded on acetylene black or charcoal have significant importance in the field of organic synthesis. Furthermore, it is noteworthy that these heterogeneous systems are characterized by high chemical atomic efficiency, which is advantageous in industrial catalysts.

## Abbreviations

CuO/AB: CuO hollow nanospheres on acetylene black; CuO/C: CuO hollow nanospheres on charcoal; EDS: Energy-dispersive X-ray spectroscopy; ICP-AES: Inductively coupled plasma atomic emission spectroscopy; TEM: Transmission electron microscopy; XRD: X-ray diffractometer.

## Competing interests

The authors declare that they have no competing interests.

## Authors’ contributions

The manuscript was written through the contributions of all authors (HW, MB, EH, JCP, HS, and KHP). All authors read and approved the final manuscript.
